# Effect of different chelators on the push-out bond strength of hydraulic cements in retrograde obturation

**DOI:** 10.34172/joddd.41378

**Published:** 2024-09-07

**Authors:** Büşra Dağıstan-Çavuşoğlu, Sıla Nur Usta

**Affiliations:** Department of Endodontics, Gulhane Faculty of Dentistry, University of Health Sciences, Ankara, Turkey

**Keywords:** EDTA, HEDP, MTA Angelus, NeoPutty, Push-out bond strength, Ultrasonic

## Abstract

**Background.:**

This study examined the effect of ethylenediaminetetraacetic acid (EDTA) and etidronic acid (HEDP) in retrograde cavities on the bond strength of MTA Angelus and NeoPutty.

**Methods.:**

Sixty-six teeth with single roots and canals were decoronated and enlarged up to F3 using the ProTaper Universal file system. After removing the apical 3 mm within the scope of endodontic surgery procedures, retrograde cavities were prepared with ultrasonic tips. The teeth were divided into three main groups according to the irrigation solution used: saline, 17% EDTA, and 9% HEDP. Following the irrigation of retrograde cavities, each main group was further divided into two subgroups in terms of using MTA Angelus and NeoPutty as retrograde filling materials. Bond strength values of hydraulic cements were measured by the push-out test. Fracture modes were examined under a stereo microscope. Two dentin sections from each group were examined under scanning electron microscopy (SEM) to observe dentinal tubules. Two-way ANOVA and post hoc Tukey tests were used to analyze the data.

**Results.:**

Irrigation solutions similarly affected the bond strength values of hydraulic cements (*P*=0.115). MTA Angelus showed significantly higher values than NeoPutty in all the solution groups (*P*=0.34). Adhesive and cohesive fracture modes were mostly observed in the MTA Angelus and NeoPutty groups, respectively.

**Conclusion.:**

EDTA, HEDP, and saline had a similar effect on the bond strength of hydraulic cements. The higher bond values of MTA Angelus compared to NeoPutty could support its safe use in endodontic surgery procedures.

## Introduction

 The success of root canal treatment depends on treating and/or preventing the formation of apical periodontitis by chemomechanical debridement of the root canal system to remove bacteria and their products.^1^ Although the success rate of root canal treatments in less than five years has been reported to be high, factors such as unretrievable broken files, persistent periradicular infections, and calcified root canals may jeopardize complete disinfection.^2^ Accordingly, endodontic root-end surgery can be performed due to its successful outcomes in such circumstances.^3^

 Root-end surgery encompasses a hermetic retrograde obturation of the root-end cavity following a minimum 2-mm resection of the apical third to minimize bacterial leakage from the canals.^4^ In this sense, placing an ideally biocompatible root-end filling material with enhanced sealing ability is essential for successful outcomes.^5^ In particular, hydraulic cements are preferred due to their superior benefits for retrograde obturation. One of these materials, MTA Angelus (Angelus Indústria de Produtos Odontológicos S/A, Londrina, PR, Brazil), is generally used as the material of choice for root-end filling since it has most of the properties of an outstanding obturation material.^6^ In the literature, compatible results of MTA Angelus have been demonstrated in terms of bond strength and sealing ability.^7,8^ However, some drawbacks regarding the standardization of mixing and handling encourage the production of new materials.^9^ In recent years, NeoPutty (NuSmile, Houston, TX, USA) has been introduced to the dental markets as a premixed format of hydraulic cements that contains tantalum oxide, tricalcium silicate, calcium aluminate, dicalcium silicate, tricalcium aluminate, and calcium sulfate.^10^ According to the manufacturers, NeoPutty has lower solubility, dimensional expansion and adequate radiopacity.^11^ Additionally, the bioactive and biocompatible properties of NeoPutty allow it to be used in multi-purpose root and pulp treatments.^12,13^ In this sense, the compatible features of NeoPutty compared to MTA led to its use in root-end surgery procedures.^10,12,14^

 A proper physical and biological structure must be provided to achieve good bonding between the root-end cavity and filling material. In particular, smear layer formation can alter the bond strength of root-end filling materials to dentin during root-end surgery applications. Ethylenediaminetetraacetic acid (EDTA) is frequently used to remove the inorganic components of the smear layer in routine endodontic practices.^15^ However, alternative chelating agents are being investigated since EDTA has low biocompatibility, is insufficient to remove the smear layer in the apical region of the root, and reduces the effectiveness of sodium hypochlorite (NaOCl) when used in combination.^16,17^ In this context, etidronic acid (1-hydroxyethylidene-1,1-bisphosphonate or HEDP) is increasingly regarded as a reliable chelating agent due to its adequate calcium chelation capacity and biocompatible structure. Unlike other chelators such as EDTA or citric acid, HEDP does not affect the antimicrobial/antibiofilm and tissue-dissolving properties of NaOCl when used in combination.^18,19^ Additionally, the more controlled demineralization effect of HEDP compared to EDTA helps preserve the sound dentin structure under the smear layer.^20^ In addition to its chelating features, it also has superior proteolytic and antimicrobial activity.^21^

 Successful outcomes after root-end surgery applications entail high precision, experience, and suitable materials and techniques. In particular, minimizing the apical leakage with an advanced bonding between the root-end cavity dentin and the filling material is crucial. To our knowledge, the use of NeoPutty as a root-end filling material in contact with EDTA and HEDP has not been investigated yet. Thus, this in vitro study aimed to evaluate the effects of EDTA and HEDP on the bond strength of MTA Angelus and NeoPutty to root-end cavity dentin in endodontic surgery applications. The first null hypothesis of the study is that different solutions used in the root-end cavity would not differ in terms of affecting the bond strength values of hydraulic cements. The second null hypothesis of the study is that different hydraulic cements used in the root-end cavity would present similar bond strength values to dentin.

## Methods

###  Sample size calculation and tooth selection

 The protocol was approved by the Ethics Committee of the University (No.: 2023/12). The sample size was calculated based on a similar study in the literature^22^ with an effect size of 0.6641, type I error probability of 0.05, and a study power of 90%. Consequently, the total required number of teeth was determined at n = 60. In addition, for the scanning electron microscopy (SEM) analysis (Philips, FEI-Quanta 400 F, Netherlands), six teeth (two teeth per group) were also included. Sixty-six extracted human teeth with caries-free, single-rooted, mature apex and less than 10º curvature^23^ were collected and evaluated under a stereomicroscope for possible fractures or anatomical malformations. The exclusion criteria for the selected teeth were calcified root canals, external or internal root resorption, and apical constriction > #15 K-file (Dentsply Maillefer, Ballaigues, Switzerland). Subsequently, the periodontal tissues of the selected teeth were removed from the external root surfaces with periodontal curettes, and the teeth were stored in 0.1% thymol solution at 4 ºC until used.

###  Root canal preparation and obturation and application of root-end surgery protocols 

 The teeth were decoronated, and the root lengths were adjusted to 16 ± 1 mm. The working length was determined at 1 mm short of where a #15 K-file became visible at the apical foramen. The root canals were instrumented with ProTaper Universal (Dentsply Tulsa Dental, Tulsa, OK, USA) rotary file system up to F3. Between files, the root canals were irrigated with 2 mL of 5.25%5 NaOCl (Microvem, Istanbul, Turkey). Final irrigation was performed using 5.25% NaOCl, 17% EDTA (Imicryl Ltd., Konya, Turkey), and distilled water, respectively. Afterward, root canal systems were obturated using F3 gutta-percha (Bio GP Points, Gyeonggi-do, South Korea) and AH Plus sealer (Dentsply, DeTrey Gmbh, Konstanz, Germany) with lateral condensation technique.

 Following root canal obturation, the apical 3 mm of the roots was resected under water cooling at a right angle to the long axis of the root to mimic endodontic surgical procedures. Root-end preparations were carried out using ultrasonic tips (E10D, Woodpecker Co., LTD, Guangxi, China). Then, the teeth were divided into three main groups randomly (https://www.randomizer.org/) in terms of the chelating agents as follows: 17% EDTA (n = 20, Imicryl Ltd., Konya, Turkey), 9% HEDP (n = 20, Dual Rinse, Medcem, Weinfelden, Switzerland), and 0.9% saline (n = 20). Root-end cavities were rinsed with 5 mL of each solution for 5 minutes.^22^

 Three main groups were then divided into two subgroups in terms of root-end filling material as follows: MTA Angelus (n = 10, Angelus Indústria de Produtos Odontológicos S/A, Londrina, PR, Brazil) and NeoPutty (n = 10, NuSmile, Houston, TX, USA). Hydraulic cements were prepared and placed into root-end cavities according to the manufacturer’s instructions. The samples were incubated under 95% relative humidity at 37 °C for one week to mimic the clinical environment.

###  Effect of chelators on the bond strength of hydraulic cements and analysis of failure modes

 One week later, the teeth were embedded in acrylic molds. Two slices of each tooth with ~1 mm thickness were obtained from the apicocoronal direction (IsoMet 1000, Buehler, IL, USA). Slices were placed in a universal testing machine (Lloyd LRX, Lloyd Instruments Ltd., Fareham, UK). A continuous load was applied to the center of the tested cement using a stainless-steel cylindrical plunger, measuring 0.7 mm^24^ in diameter, mounted on the machine. The push-out force was applied with a 1 mm/min crosshead speed in the coronoapical direction until bond failure occurred between the root dentin and the cement. The bond strength value was recorded in Newton (N) and calculated by converting these forces to Megapascals (MPa).

 The fractured surfaces were examined under × 12 magnification under a stereomicroscope (Leica M165C, Leica Mycrosystems Ltd, Wetzlar, Germany). The failure modes were classified as adhesive, cohesive, and mixed.

###  SEM and statistical analysis

 Six teeth (two from each solution group) were examined with SEM to observe the dentinal tubules (B.D.Ç.). In this context, dentin discs were vacuum-dried and coated with gold under 20 kV current; then, the samples were examined under × 5000 magnification, and images were taken. SEM images were evaluated by another researcher (S.N.U.) who was blinded to the groups.

 Data were analyzed using SPSS 26 (Chicago, IL, USA). The Kolmogorov-Smirnov and Levene tests were used to check the normal distribution and homogeneity of data, respectively. Two-way ANOVA and post hoc Tukey tests were used to evaluate the effect of different irrigation solutions on the bond strength of MTA Angelus and NeoPutty. The level of significance was set at *P* < 0.05.

## Results

**Figure 1:**
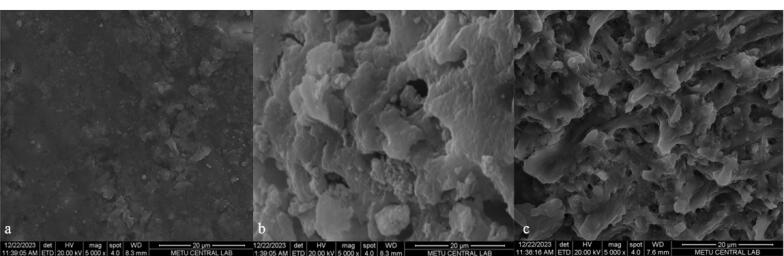
SEM images of solution groups (a) saline, (b) EDTA, (c) HEDP

 SEM analysis revealed that while dentinal tubules were covered with the smear layer in the saline group, EDTA and HEDP were associated with smear layer removal efficiency (Figure 1).

 Regardless of the root-end filling material, although higher bond strength values were observed in the saline group, no statistically significant difference was found between EDTA, HEDP, and saline groups (*P* = 0.115). The push-out bond strength values of MTA Angelus were significantly higher than NeoPutty in all irrigation solution groups (*P* = 0.34). Table 1 shows the main and standard deviations of bond strength values based on groups.

**Table 1:** Mean ± standard deviation (SD) of the push-out bond strength values of the groups in terms of solutions and hydraulic cements

	**n**	**MTA Angelus**	**NeoPutty**	**Comparison ** ***P*** ** value**
Saline	20	25.06 ± 9.15^a,1^	21.71 ± 4.80^b,1^	0.115
EDTA	20	20.54 ± 11.63^a,1^	15,50 ± 5.08^b,1^	
HEDP	20	24.99 ± 10.64^a,1^	19.29 ± 6.18^b,1^	
Comparison *P* value		0.034		

Different superscript lowercase letters in the same row indicate a statistically significant difference (*P* < 0.05). The same superscript numbers in the same column indicate no statistically significant difference (*P* > 0.05).

**Figure 2:**
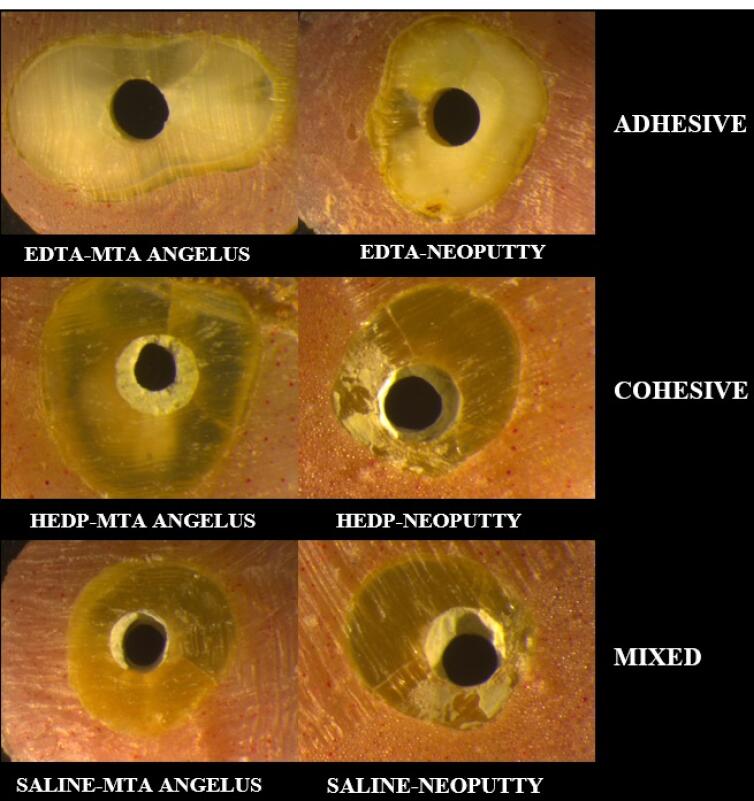
Representative images of failure modes based on groups

Figure 2 shows the failure modes.Cohesive, mixed, and adhesive failure modes were observed more frequently in the EDTA, HEDP, and saline groups, respectively. Moreover, adhesive and mixed failure modes were most common in the MTA Angelus group, while cohesive and mixed fracture modes were observed in the NeoPutty group. Table 2 presents the number and percentage of failure modes in each group.

**Table 2:** Number and percentage of failure types for each group

**Groups**	**N**	**Failure types (n (%))**		
		**Adhesive**	**Cohesive**	**Mixed**
EDTA-MTA Angelus	20	6 (30%)	6 (30%)	8 (40%)
HEDP-MTA Angelus	20	12 (60%)	2 (10%)	6 (30%)
Saline-MTA Angelus	20	4 (20%)	10 (50%)	6 (30%)
EDTA-NeoPutty	20	4 (20%)	8 (40%)	8 (40%)
HEDP-NeoPutty	20	6 (30%)	8 (40%)	6 (30%)
Saline-NeoPutty	20	6 (30%)	8 (40%)	6 (30%)

## Discussion

 Clinical studies can demonstrate the outcomes of root-end surgery procedures effectively.^25^ However, well-designed in vitro studies are also needed to assess the possible effects of newly developed materials recommended for use in root-end cavities. In this sense, using NeoPutty as a relatively new material has not been investigated in root-end surgery applications. Moreover, possible interactions between NeoPutty and chelators in the apical thirds of teeth have not also been indicated. Therefore, this study aimed to signify the bond strength of NeoPutty in retrograde obturation in contact with EDTA and HEDP, compared with MTA Angelus. While the first null hypothesis was accepted, the second one was rejected since MTA Angelus and NeoPutty showed statistically significant differences.

 A strong bond between retrograde obturation material and dentin is important to enhance the sealing ability and resist dislodgement forces.^26^ The filling material used and the physicochemical environment of the root-end cavity space resulting from solutions and blood contamination can be considered influential factors for adhesion.^22,27^ Accordingly, the chemical structure and setting properties of hydraulic cements may be affected by chelators. In this sense, conflicting results have been reported in the literature regarding the smear layer removal for a strong bond of hydraulic cements to the dentin.^22,28^ Since study designs and materials used have unique properties, diversities in the results prevent reaching a definite conclusion.

 In this study, solutions did not differ in terms of affecting the bond strength of MTA Angelus and NeoPutty in root-end cavities, as in previous studies.^22,29^ However, the values were relatively higher and lower in saline and EDTA groups, respectively, which can be explained by the fact that EDTA was shown to hinder the hydration mechanism by chelating calcium ions released from hydraulic cements and reducing their chemical adhesion to dentin.^30^ Another reason for reduced values in the EDTA group could be the impaired surface hardness of hydraulic cements in an acidic environment.^31^ Valencia et al^32^ reported the improved bond strength of Portland cement when EDTA was used for smear layer removal. They attributed this result to the decreased contact surface at the material‒dentin interface and the intratubular formation of tail-like structures. The different results may be interpreted by the differences in exposure times and amounts of exposure to the solution, concentrations, material properties, and the investigated tooth part.

 This study indicated similar values between HEDP and saline groups with no detrimental effects. One possible explanation could be the enhanced hydration of hydraulic cements that have been demonstrated by Neelakantan et al^33^ through the formation of a highly crystalline surface and a high release of calcium in samples of hydraulic cements after being wrapped in a gauze soaked in a mixture of 6% NaOCl and 18% HEBP. Moreover, Rebolloso de Barrio et al showed higher bond strength values of MTA after one day of exposure to NaOCl + HEDP.^34^ Ulusoy et al^35^ also reported higher dislodgement resistance of MTA in the HEDP group compared to EDTA. However, direct comparisons cannot be made since there is no information regarding the effect of HEDP on the adhesion of the MTA Angelus and NeoPutty in root-end cavities.

 Although NeoPutty is suggested as a root-end filling material,^10^ the bond strength values were significantly lower compared to MTA Angelus, which can be explained by the different diffusion capacities of these cements into dentinal tubules. Another possible reason could be the particle size difference between MTA Angelus and NeoPutty. In this context, it was considered that MTA Angelus may contain larger particles due to the presence of bismuth oxide. Accordingly, larger particle sizes could help sustain higher loads.^36^ However, interestingly, MTA Angelus groups mainly exhibited adhesive and mixed types of failures. Although the large particle size of the material provides good adhesion in the superficial dentin, it is thought that it may have caused massive displacement as it prevented deep invasion into the dentinal tubules. In addition, it should also be remembered that the analysis of failure modes is not the sole criterion for assessing the bond strength.^37^

 Although the push-out test is widely accepted and used to assess the bond strength of dentin to different materials, this method deserves attention,^38^ especially concerning the diameter of the plunger tips and samples used, the properties of the materials, and the applied force that might lead to different results across studies. In this sense, test set-up and sample sizes were determined according to the study performed by Chen et al^24^ to minimize the limitations. Moreover, using single-rooted tooth apical root sections allowed a highly standardized and reproducible protocol with samples of a constant thickness.^28^ Finally, since bond strength values can be interpreted within the scope of smear layer removal, observing dentinal tubules with SEM has implied a more accurate and effective evaluation process.

## Conclusion

 Comparable effects of HEDP with EDTA and saline support its use as a root-end filling material. NeoPutty showed lower push-out bond strength values than MTA Angelus. Future well-designed studies are needed to better evaluate the interaction between NeoPutty and dentin along with irrigation solutions.

## Acknowledgments

 The authors would like to thank Health Sciences University’s Scientific Research Committee for supporting this study (grant no: 2023/025).

## Competing Interests

 None.

## Ethical Approval

 The protocol of this study was approved by the Ethics Committee of the University of Health Sciences (Code: 2023/12).
